# Unique Finding of a Primary Central Nervous System Neuroendocrine Carcinoma in a 5-Year-Old Child: A Case Report

**DOI:** 10.3389/fnins.2022.810645

**Published:** 2022-03-23

**Authors:** Natalia Stepien, Christine Haberler, Sarah Theurer, Maria-Theresa Schmook, Carola Lütgendorf-Caucig, Leonhard Müllauer, Johannes Gojo, Amedeo A. Azizi, Thomas Czech, Irene Slavc, Andreas Peyrl

**Affiliations:** ^1^Department of Pediatric and Adolescent Medicine, Medical University of Vienna, Vienna, Austria; ^2^Comprehensive Center for Pediatrics, Medical University of Vienna, Vienna, Austria; ^3^Division of Neuropathology and Neurochemistry, Department of Neurology, Medical University of Vienna, Vienna, Austria; ^4^Institute of Pathology, University of Duisburg-Essen, Essen, Germany; ^5^Department of Biomedical Imaging and Image-Guided Therapy, Medical University of Vienna, Vienna, Austria; ^6^MedAustron Ion Therapy Center, Wiener Neustadt, Austria; ^7^Department of Pathology, Medical University of Vienna, Vienna, Austria; ^8^Department of Neurosurgery, Medical University of Vienna, Vienna, Austria

**Keywords:** pediatric brain tumor, neuroendocrine carcinoma (NEC), primary CNS tumor, rare entities, neuroendocrine tumors

## Abstract

Neuroendocrine tumors (NETs) are rare neoplasms predominantly arising in the gastrointestinal-tract or the lungs of adults. To date, only ten cases of primary central nervous system (CNS) NETs have been reported, with just three of them describing a neuroendocrine carcinoma (NECA) and none occurring in a child. We report on a previously healthy 5-year-old boy, who presented with headaches, nausea and vomiting, and was diagnosed with a left cerebellar solid mass with a cystic component. After gross-total resection, histology revealed a neuroendocrine carcinoma. Molecular analysis of the tumor tissue showed a *KRAS*-splice-site mutation (c451-3C > T). The *KRAS*-mutation was discovered to be a maternal germline mutation, previously described as likely benign. After extensive search for an extracranial primary tumor, including Ga-68 DOTANOC-PET-CT, the diagnosis of a primary CNS NECA was established, and proton irradiation was performed. Unfortunately, the patient developed an in-field recurrence just 5 weeks after the end of radiotherapy. The tumor was re-resected with vital tumor tissue. Six cycles of chemotherapy were initiated, consisting of cisplatin, carboplatin, etoposide and ifosfamide. The patient remains disease free 22 months after the end of treatment, supporting the beneficial effect of platinum- and etoposide-based chemotherapy for this tumor entity.

## Introduction

Tumors of the central nervous system (CNS) are the most frequent type of solid neoplasms in children (Ostrom et al., 2020). However, they are comprised of more than hundred different entities, and while the more frequent ones are considered to be a rare disease (i.e., with an incidence of <1:2,000), some entities are only described anecdotally (Richter et al., 2015). We present the case of a primary CNS neuroendocrine carcinoma, a tumor entity that has been recently described in a few case reports on adult patients (Tamura et al., 2014; Liu et al., 2016; Reed et al., 2019), but not yet in the pediatric population.

## Case Report

**FIGURE 1:**
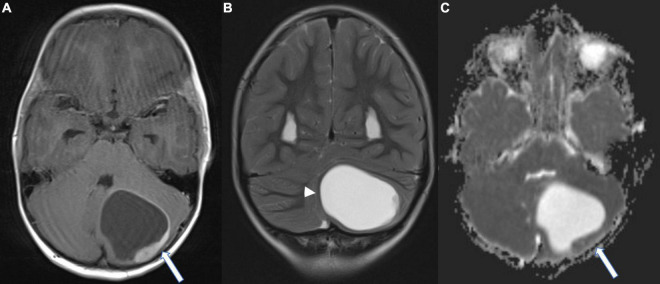
Magnet resonance (MR) images at the time of diagnosis; **(A)** Axial T1-weighted contrast-enhanced image showing a left cerebellar cystic mass with a peripheral contrast enhancing component (arrow); **(B)** Coronal T2-weighted image. Note the infratentorial midline shift due to the mass effect of the cystic component (arrowhead). **(C)** Axial diffusion-weighted image (ADC-Map) demonstrating low ADC values of the solid component (arrow), suggestive of high cellular density.

We report on a previously healthy 5-year-old boy, who had been suffering from headaches, accompanied by nausea and intermittent vomiting for 4 weeks prior to diagnosis. Magnet resonance imaging (MRI) of the head showed a left cerebellar cystic mass and hydrocephalus with signs of transependymal cerebrospinal fluid (CSF) diapedesis. The appearance on MRI (Figures 1A,B) with comparatively low apparent diffusion coefficient (ADC) values within the solid component directed toward higher cellularity (Figure 1C). Surgical resection of the tumor mass was performed. Postoperative MRI did not show any signs of residual tumor nor metastasis.

**FIGURE 2:**
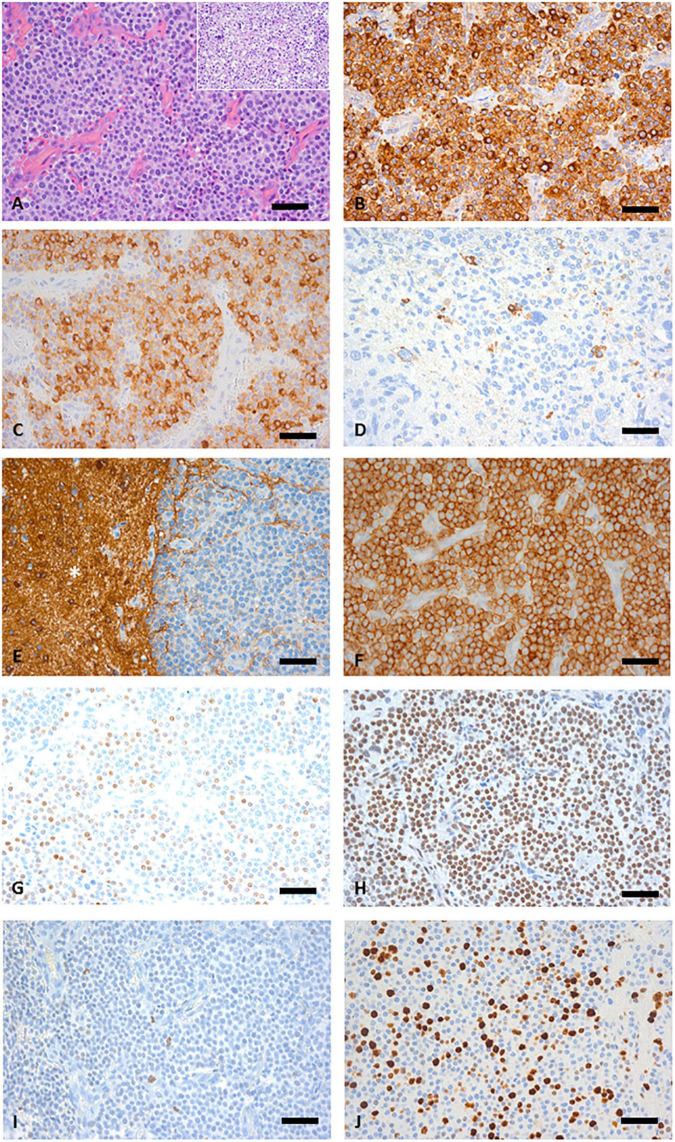
**(A)** Hematoxylin and eosin (HE) sections of the biopsy specimen showing a highly cellular tumor growing in a sheet-like pattern separated by fibrovascular septa, inset depicts an area with pleomorphic tumor cells. **(B)** Staining with pan-cytokeratin [Lu-5] antibody confirms an epithelial origin. **(C)** Anti-Chromogranin A staining is positive in most tumors cells. **(D)** Only few scattered synaptophysin positive tumor cells were detectable. **(E)** NCAM staining was negative in the tumor tissue but the asterisk (*) indicates positive NCAM staining in the adjacent white matter. **(F)** Widespread SSTR2 expression. **(G)** NeuN showing a moderate intensity in a fraction of tumor cell nuclei. **(H)** Widespread expression of OTX2. **(I)** pHH3 reveals frequent mitotic figures. **(J)** Ki67 proliferation; 35.4%. **(A–J)** Original magnification x400, scale bar represents 50 μm.

Histologic analysis of the tumor material revealed an epithelial neoplasm composed of predominantly small uniform cells with focally moderately anaplastic elements (Figure 2A). Immunohistochemically, expression of pancytokeratin (Figure 2B), CK8, CK18 CK19, and EMA was detectable. No immunopositivity was observed for CK7, CK20, and p63. Chromogranin A was expressed in the majority of tumor cells (Figure 2C) and few cells displayed synaptophysin, (Figure 2D) pointing toward a neuroendocrine differentiation, yet CD56 was negative within the tumor cells (Figure 2E). No serotonin and CD117 immunoreactivity could be detected, whereas SSTR2 (Figure 2F) and SSTR5 expression was present. To exclude other CNS and non-CNS tumors a broad panel of immunohistochemical stainings was performed (Table 1). A smaller fraction of cells showed a moderately intense expression of NeuN. OTX2 was moderately intense expressed in the majority of tumor cells (Figure 2H). The anti-pHH3 staining (Figure 2I) revealed up to 22 mitoses per mm^2^ and the Ki-67 proliferation index was 35.4% (Figure 2J). Due to the combined expression of epithelial and neuroendocrine markers the tumor was classified as neuroendocrine carcinoma.

**TABLE 1:** All antibodies/company used and the respective results in the tumor cells.

Antibody	Company, clone	Tumor cells
Pancytokeratin	Dako/Agilent; Lu-5	Pos
CK5/6	Dako/Agilent; D5/16 B4	Neg
CK7	Dako/Agilent; DOV-TL 12/30	Neg
CK8	BD Biosciences; CAM 5.2	Pos
CK18	Dako/Agilent; DC 10	Pos
CK19	Dako/Agilent; RCK108	Pos
CK20	Dako/Agilent; Ks 20.8	Neg
p63	Ventana/Roche; 4A4	Neg
EMA	Dako/Agilent; E29	Pos
Chromogranin A	Dako/Agilent; DAK-A3	Pos
CD56	Monosan; 123C3	Neg
Synaptophysin	Dako/Agilent; DAK-SYNAP	Scattered cells pos
Serotonin	Dako/Agilent; 5HT-H209	Neg
SSTR2	Abcam; UUMB1	Pos
SSTR5	Abcam; UMB4	Pos
CD117	Dako/Agilent; polyclonal	Neg
Pit-1	Santa Cruz; D-7	Neg
SF1	R&D Systems; N1665	Neg
ACTH	Dako/Agilent; 02A3	Neg
TTF1α	Ventana/Roche; 8G7G3/1	Neg
GFAP	Dako/Agilent; polyclonal	Neg
Olig2	IBL; polyclonal	Neg
S100	Dako/Agilent; polyclonal	Neg
Smooth muscle actin	Dako/Agilent; 1A4	Neg
Vimentin	Dako/Agilent; V9	Neg
MAP2	Merck/Millipore; AP20	Neg
Neurofilament H phosphorylated	Covance; SMI-31	Neg
Neurofilament H non-phosphorylated	Covance; SMI-32	Neg
NeuN	Merck/Millipore; A60	Some cells positive
Lin28A (A177)	Cell Signaling Technology; polyclonal	Neg
CRX (A-9)	Santa Cruz Biotechnology	Neg
CD99	BioGenex; EP8	Neg
BCoR (C-10)	Santa Cruz Biotechnology	Neg
NUT	Cell Signaling Technology; C52B1	Neg
OCT-3/4 (C-10)	Santa Cruz Biotechnology	Neg
H3 p.K28me3	Invitrogen; polyclonal	Pos
H3 p.K28M	Abcam; EPR18340	Neg
SMARCB1	BD Biosciences; 25/BAF47	Pos
SMARCA4	Abcam; EPNCIR111A	Pos
SDHB	Abcam; (21A11AE7)	Pos
OTX2	ThermoFisher; 1H12C4B5	Pos
MelanA	Biocare Medical; HMB45/MART-1/Tyrosinase	Neg
Ki67	Dako/Agilent; MIB-1	35% proliferation
pHH3	Merck/Sigma-Aldrich; polyclonal	≥2 mitoses/mm^2^
MSH2	Cell Marque; G219-1129	Pos
MSH6	Cell Marque; 44	Pos
MLH1	Ventana/Roche; M1	Pos
PMS2	Cell Marque; EPR3947	Pos
ALK	Zytomed; 1A4;	Neg
NTRK	Abcam; EPR17341	Neg

Further molecular analysis was performed using the Ion AmpliSeq Cancer HotSpot Panel v2 and Oncomine Comprehensive Assay v3 (both: Thermo Fisher Scientific, Waltham, MA, United States), showing a splice site mutation in *KRAS* (c451-3C > T) with an allele frequency of 50%, which was later confirmed as a germ-line mutation, inherited from the patient’s asymptomatic mother. No alterations were detected in *MEN1* and *RET*. An extensive search for a primary tumor outside the CNS was initiated. F-18-FDG PET-CT of the cervical, thoracal, and abdominal area did not show signs of increased uptake. Ga-68-DOTANOC PET-CT from the head to the symphysis demonstrated a slightly elevated uptake in the area of tumor resection without any other areas of increased uptake. In addition, ultrasound of the thyroid and abdomen as well as a capsule endoscopy were performed. None of these examinations showed signs of an extracranial primary tumor. Neuron-specific enolase was slightly elevated (25.6 μg/L), other markers of neuroendocrine tumors (NETs), such as insulin, glucagon, vasoactive intestinal peptide (VIP) were within the normal range. Therefore, the diagnosis of a primary neuroendocrine carcinoma (NECA) of the CNS was established and focal proton therapy initiated 5 weeks after diagnosis (54 Gy/60 Gy (PTV1/PTV2) relative biologic effectiveness in 30 fractions).

**FIGURE 3:**
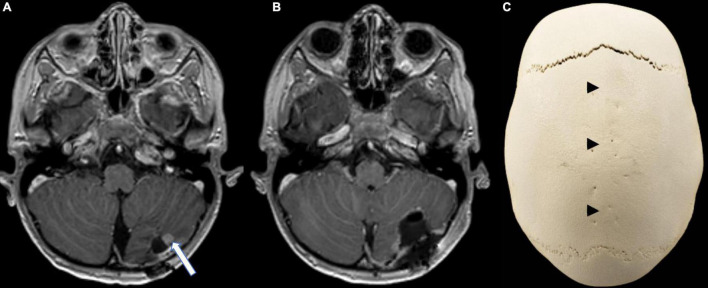
Follow-up imaging. **(A)** Axial contrast-enhanced T1-weighted MR image showing a contrast enhancing nodule (arrow) at the infero-lateral border of the resection cavity, strongly suggestive of local recurrence. **(B)** Axial contrast-enhanced T1-weighted MR image after en-bloc resection of the recurrent tumor. **(C)** CT reconstruction (Volume rendering) of the skull highlighting the dolichocephaly and the premature synostosis of the sagittal suture (arrow heads) when compared to the open sutures.

The MRI 5 weeks after completion of radiotherapy showed local recurrence of the primary tumor within the field of irradiation (Figure 3A). En-bloc re-resection was performed and biopsies from the surrounding tissue were taken (Figure 3B). Vital tumor cells of the previously diagnosed NECA were found within the resected tissue, while the biopsies from the surrounding areas were negative for tumor cells. Systemic cytotoxic therapy with a total of six cycles was given (cumulative doses: cisplatin 200 mg/m^2^, carboplatin 2,400 mg/m^2^, etoposide 2,400 mg/m^2^, ifosfamide 12,000 mg/m^2^). At the end of chemotherapy, no sign of recurrence or metastases was detected in MRI. Even 22 months after the end of chemotherapy there is still no sign of recurrence, the patient is in good clinical condition, attending school and participating in daily life activities without any limitations.

## Discussion

Neuroendocrine carcinomas (NECAs) are a subgroup of neuroendocrine tumors (NETs) originating from neuroendocrine cells, defined by increased proliferation markers (Ki-67 index > 20%) and loss of differentiated histomorphology (DeLellis, 2001; Oronsky et al., 2017). These widely dispersed cells are characterized by the presence of endocrine and neuronal features and can give rise to NETs in virtually all organs. However, in adults the majority of primaries arise in the gastrointestinal tract (62–67%) and the lungs (22–27%) (DeLellis, 2001; Oronsky et al., 2017).

Neuroendocrine tumors (NETs) are extremely rare in the pediatric population, so that the incidence can only be estimated, with a range from 1 to 5 per 1,000,000 people with hardly any case reported in children below the age of 10 (Navalkele et al., 2011; Diets et al., 2017; Stawarski and Maleika, 2020). The most common site of occurrence in the pediatric population is the appendix (Diets et al., 2017). However, so far, no case of primary CNS NET has been described in the pediatric population.

While most NETs occur sporadically, certain genetic syndromes predispose the development, including multiple endocrine neoplasia type 1 and 2 (MEN-1 and MEN-2) (Walls, 2014), neurofibromatosis (Gut et al., 2015), tuberous sclerosis (Gut et al., 2015) and von Hippel-Lindau disease (Rednam et al., 2017). Occurrence at young age and/or positive family history should prompt for genetic counseling and testing. Our patient showed neither clinical characteristics of these syndromes, nor was MEN 1/2 detected in the genetic analysis.

Irrespective of the primary site, NETs share some histologic characteristics and can be divided into differentiated NETs and NECAs. Well differentiated NETs are characterized by “organoid” or neuroendocrine shaped arrangement of tumor cells producing neurosecretory granules, intensely reacting to neuroendocrine markers, including synaptophysin and chromogranin A. Staining intensity with neuroendocrine markers in NECA can be less, but is by definition present. In contrast to NETs, proliferation index with Ki-67 is >20% in NECAs. Histomorphology in NECA is undifferentiated with solid or diffuse growth and nuclear atypia (Klimstra et al., 2010; Oronsky et al., 2017). Previous case reports of primary CNS NETs include seven cases of differentiated NETs (Porter et al., 2000; Deshaies et al., 2004; Ibrahim et al., 2010; Hood et al., 2014; Liu et al., 2016; Vernieri et al., 2016; Hakar et al., 2017) and three cases of NECAs (Tamura et al., 2014; Liu et al., 2016; Reed et al., 2019). Primary CNS NECAs were described to stain positive for neuroendocrine markers, including chromogranin and synaptophysin, and negative for the glial marker GFAP. This also was the case in our patient, who was positive for CK8, CK18, and CK19, additionally, while being negative for CK7 and CK20, vimentin, S100, Olig2, and MAP2. In contrast to a previous case report of a neuroendocrine tumor in the brain, which was slightly CD56 positive (Tamura et al., 2014), we could not detect CD56 expression. Somatostatin receptor (SSTR) status was not reported in other cases of CNS NECA, however, 100% of cells in our specimen were positive for SSTR-2, and 80% were positive for SSTR-5. The unusual pathological and immunohistochemical features of this tumor require careful delineation from other pediatric CNS tumor types. The expression of chromogranin A and synpatophysin raises the suspicion of a CNS embryonal tumor, particularly medulloblastoma, atypical teratoid/rhabdoid tumor, CNS neuroblastoma, FOXR2-activated or CNS embryonal tumor NEC/NOS. However, none of these tumors displays expression of cytokeratin throughout the whole tumor tissue. On the other hand, tumors of the choroid plexus are characterized by widespread cytokeratin expression. Yet, these tumors show a papillary architecture, which was not present in our case and no neuroendocrine differentiation. Furthermore, an ectopic pituitary adenoma was excluded due to lack of Pit1, SF1, and ACTH expression. Thus, the morphological and immunohistochemical features do not support the diagnosis of any other CNS tumor entity. Interestingly, the tumor showed expression of OTX2, which plays an important role in the development of the midbrain-hindbrain region (Di Giovannantonio et al., 2014) and is expressed in medulloblastomas (de Haas et al., 2006).

Because of the rarity of primary intracranial neuroendocrine carcinomas, it is obligatory to perform a thorough screening for an extracranial primary before establishing the diagnosis. In addition to the standard screening methods, including MRI/CT of the chest and abdomen, thyroid ultrasound, gastro- and colonoscopy, chromogranin and further symptom based biochemical testing, the recently developed method of receptor-based PET-CT/MRI adds more sensitivity to the already available functional imaging (Ambrosini et al., 2008; Naswa et al., 2012; Raphael et al., 2017).

While for some low-grade NETs surgical resection is sufficient, NECAs necessitate further chemotherapy, which in most cases is platinum-based (Oronsky et al., 2017; Rinke and Gress, 2017). However, all case reports on primary CNS NECAs reported radiation therapy as their first choice (Tamura et al., 2014; Reed et al., 2019). Considering the beneficial effect of radiotherapy in most pediatric CNS high-grade malignancies, proton therapy was the first-line treatment. Unfortunately, the tumor recurred very rapidly 5 weeks after the end of radiation within the irradiation field, necessitating re-resection and systemic cytotoxic therapy.

Besides the previously mentioned genetic syndromes, loss of RB1 and p53 function is one of the molecular characteristics described in various NECA locations (Kawasaki et al., 2020; McNamara et al., 2020). Further mutations differ depending on the localization and grade, with frequent alterations in *PIK3CA/PTEN*, *BRAF*, and *KRAS* (Sahnane et al., 2015; Olevian et al., 2016; Vijayvergia et al., 2016; Oronsky et al., 2017; Von Arx et al., 2019). No genetic information was available on the previously published primary CNS NECAs (Tamura et al., 2014; Liu et al., 2016; Reed et al., 2019), however, the tumor material of our patient was extensively analyzed, resulting in the detection of a germ-line splice site mutation in *KRAS* (exon5: c.451-3C > T), an alteration previously not described in NECAs. This variant is located in an alternate transcript (*KRAS-A*) of *KRAS* and likely benign, since it was only identified in an unaffected parent of a patient with Noonan syndrome. This is further supported by the finding of the same variant in our patient’s mother, who did not suffer from any malignancy and the unremarkable family history regarding oncologic diseases. Interestingly, craniosynostosis and scaphocephaly was found in our patient (Figure 3C). Most cases of craniosynostosis develop sporadically, especially sagittal synostosis, where genetic alterations can be found in less than 1% of cases (Wilkie et al., 2017). Several molecular alterations have been identified as important in the development of craniosynostosis, including ERF, a regulator in the RAS-MAP-kinase pathway, as well as KRAS itself, mostly within syndromic cases (Addissie et al., 2015; Wilkie et al., 2017). Again, our patient did not show any clinical signs of Noonan or similar RASopathies, and it remains unclear whether the *KRAS* mutation detected in our young patient is to be considered as a polymorphism, not involved in the development of neither the craniosynostosis nor the neuroendocrine carcinoma, or if it played a role in the disease development.

## Conclusion

To the best of our knowledge, primary CNS neuroendocrine tumors have not yet been described previously in children. Reports about primary CNS NECAs appeared only recently and covered just the adult population. Extensive screening is necessary to exclude any extracranial primary tumor before establishing this diagnosis. The aggressiveness of this tumor has been demonstrated by its rapid in-field recurrence after irradiation. Similar to extracranial NECAs, platinum-based chemotherapy seems to be the therapy of choice.

## Data Availability Statement

The original contributions presented in the study are included in the article/Supplementary Material, further inquiries can be directed to the corresponding authors.

## Ethics Statement

Ethical review and approval was not required for the study on human participants in accordance with the local legislation and institutional requirements. Written informed consent to participate in this study was provided by the participants’ legal guardian/next of kin. Written informed consent was obtained from the minor(s)’ legal guardian/next of kin for the publication of any potentially identifiable images or data included in this article.

## Author Contributions

NS, JG, IS, and AP: study concept and design. NS, CH, ST, M-TS, CL-C, LM, JG, AA, TC, IS, and AP: acquisition of data. NS, CH, ST, M-TS, LM, JG, AA, TC, IS, and AP: analysis and interpretation of data and critical revision of the manuscript for important intellectual content. NS and AP: drafting of the manuscript. JG, IS, and AP: study supervision. All authors contributed to the article and approved the submitted version.

## Conflict of Interest

The authors declare that the research was conducted in the absence of any commercial or financial relationships that could be construed as a potential conflict of interest.

## Publisher’s Note

All claims expressed in this article are solely those of the authors and do not necessarily represent those of their affiliated organizations, or those of the publisher, the editors and the reviewers. Any product that may be evaluated in this article, or claim that may be made by its manufacturer, is not guaranteed or endorsed by the publisher.

## Supplementary Material

Supplementary Figure 1 Pre-existing cerebellar tissue shows synaptophysin (arrow) and NCAM expression (asterisk).
