# Supplementary figures and images for: Unique Finding of a Primary Central Nervous System Neuroendocrine Carcinoma in a 5-Year-Old Child: A Case Report
Source: Front Neurosci. 2022 Mar 23;16:810645. doi: 10.3389/fnins.2022.810645 (PMC8984181; doi:10.3389/fnins.2022.810645)

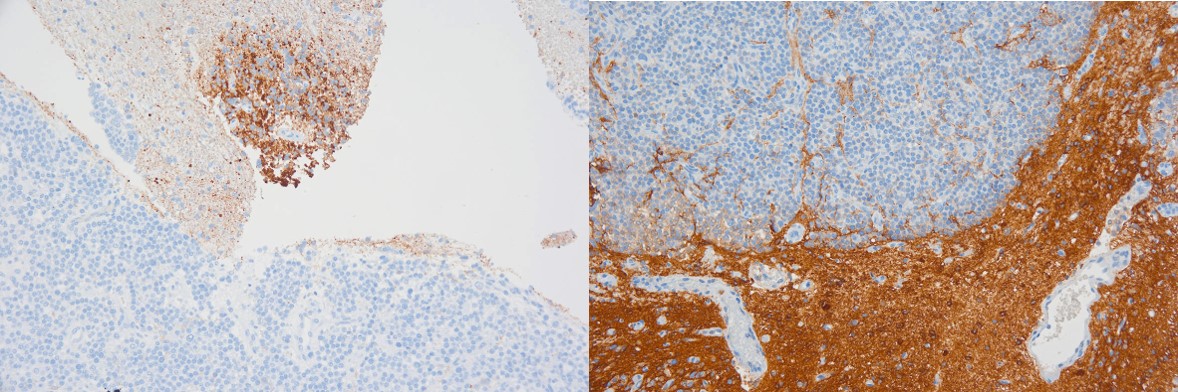

Supplement: Supplementary Figure 1 — Pre-existing cerebellar tissue shows synaptophysin (arrow) and NCAM expression (asterisk). [file Image_1.JPEG]
